# Genetic Association of* MMP10*,* MMP14*, and* MMP16* with Dental Caries

**DOI:** 10.1155/2017/8465125

**Published:** 2017-02-28

**Authors:** D. D. Lewis, J. R. Shaffer, E. Feingold, M. Cooper, M. M. Vanyukov, B. S. Maher, R. L. Slayton, M. C. Willing, S. E. Reis, D. W. McNeil, R. J. Crout, R. J. Weyant, S. M. Levy, A. R. Vieira, M. L. Marazita

**Affiliations:** ^1^Department of Human Genetics, Graduate School of Public Health, University of Pittsburgh, Pittsburgh, PA, USA; ^2^Department of Biostatistics, Graduate School of Public Health, University of Pittsburgh, Pittsburgh, PA, USA; ^3^Center for Craniofacial and Dental Genetics, School of Dental Medicine, University of Pittsburgh, Pittsburgh, PA, USA; ^4^Department of Oral Biology, School of Dental Medicine, University of Pittsburgh, Pittsburgh, PA, USA; ^5^Department of Pharmaceutical Sciences, School of Pharmacy, University of Pittsburgh, Pittsburgh, PA, USA; ^6^Department of Psychiatry, School of Medicine, University of Pittsburgh, Pittsburgh, PA, USA; ^7^Department of Mental Health, Johns Hopkins Bloomberg School of Public Health, Johns Hopkins University, Baltimore, MD, USA; ^8^Department of Pediatric Dentistry, School of Dentistry, University of Washington, Seattle, WA, USA; ^9^Division of Genetics and Genomic Medicine, Department of Pediatrics, School of Medicine, Washington University at St. Louis, St. Louis, MO, USA; ^10^Department of Medicine, School of Medicine, University of Pittsburgh, Pittsburgh, PA, USA; ^11^Clinical and Translational Science Institute, School of Medicine, University of Pittsburgh, Pittsburgh, PA, USA; ^12^Psychology, Dental Practice and Rural Health, West Virginia University, Morgantown, WV, USA; ^13^Department of Periodontics, School of Dentistry, West Virginia University, Morgantown, WV, USA; ^14^Department of Dental Public Health and Information Management, School of Dental Medicine, University of Pittsburgh, Pittsburgh, PA, USA; ^15^Department of Preventive and Community Dentistry, University of Iowa College of Dentistry, Iowa City, IA, USA; ^16^Department of Epidemiology, University of Iowa College of Public Health, Iowa City, IA, USA

## Abstract

Matrix metalloproteinases (MMPs), which degrade extracellular proteins as part of a variety of physiological processes, and their inhibitors have been implicated in the dental caries process. Here we investigated 28 genetic variants spanning the* MMP10*,* MMP14*, and* MMP16* genes to detect association with dental caries experience in 13 age- and race-stratified (*n* = 3,587) samples from 6 parent studies. Analyses were performed separately for each sample, and results were combined across samples by meta-analysis. Two SNPs (rs2046315 and rs10429371) upstream of* MMP16* were significantly associated with caries in an individual sample of white adults and via meta-analysis across 8 adult samples after gene-wise adjustment for multiple comparisons. Noteworthy is SNP rs2046315 (*p* = 8.14 × 10^−8^) association with caries in white adults. This SNP was originally nominated in a genome-wide-association study (GWAS) of dental caries in a sample of white adults and yielded associations in a subsequent GWAS of surface level caries in white adults as well. Therefore, in our study, we were able to recapture the association between rs2046315 and dental caries in white adults. Although we did not strengthen evidence that* MMPs 10*,* 14*, and* 16* influence caries risk,* MMP16* is still a likely candidate gene to pursue.

## 1. Introduction

Despite significant improvements in oral health in the U.S, dental caries still remains the most prevalent chronic disease among children. The etiology of caries is multifactorial, involving a number of environmental factors, including microbial flora and fluoride exposure. Although these environmental factors substantially contribute to the disease itself, the impact of genetic factors plays a considerable role that has been long recognized and studied [1].

Evidence of genetic contribution to variation in liability to caries has been detected in studies showing heritability estimates between 40% and 60% [2–4]. Furthermore, over the past decade, there have been several studies that have nominated candidate genes based on their known biological functions in oral health such as genes involved in enamel formation [5–8], tooth development, [8, 9], taste preference [4, 10, 11], and host defense [12–15].

Matrix metalloproteinases (MMPs) are a well-studied multigene family that belongs to the metalloproteinase class of endopeptidases which are responsible for the remodeling and degradation of extracellular matrix molecules (ECM) [16]. ECM macromolecules are essential for maintaining a cellular environment for biological processes such as embryonic development, tissue remodeling, wound healing, and angiogenesis [17]. MMPs are involved in several diseases such as cancer [18], arthritis [19], tissue ulceration [20], periodontitis [21, 22], early tooth development [23, 24], and dental caries [16, 25, 26].

Studies have demonstrated that MMPs are involved in dental caries lesion progression by their presence in both dentin and saliva and their active role in the dentin matrix degradation [24, 27–29]. The initiation of caries is a dynamic process of which the mineral part of the dentin is dissolved (demineralization), exposing the organic matrix to degradation by bacterial and host derived enzymes such as MMPs [30]. Subsequent to demineralization, the breakdown of the collagenous organic matrix in the dentin occurs, which is necessary for caries formation [31]. Members of the metalloproteinase family such as* MMP10*, also referred to as stromelysin-2, are capable of degrading all components of the extracellular matrix [32]. Even more, a preliminary analysis of GWAS data suggested that variation in* MMP10* may increase the risk for dental caries [8, 33], thus providing compelling evidence for further investigation.

Several members of the MMP family have been observed in cells and tissues of the dentine-pulp complex and are thought to be involved in physiological processes during early tooth development [29, 34]. Correspondingly,* MMP14* is a membrane-type 1 matrix metalloproteinase (MT1-MMP), a membrane-bound member of the MMP gene family that has been identified to be expressed on the cell surface of ameloblasts and odontoblasts of the developing tooth [35]. Additionally, studies have demonstrated delayed tooth eruption in* mmp14* deficient mice [36] and have shown that* MMP14* plays a role in the activation of the enamel matrix protein* MMP20* [37]. Likewise,* MMP16*, a member of the type I transmembrane group as well, has been suggested to be involved in tooth development by regulating ameloblast maturation and enamel formation [38]. The rationale for the selection of* MMP10*,* MMP14,* and* MMP16* was based on their biological role in tooth formation and plausible effects on caries etiology. Therefore, the aim of this study is to determine if variants spanning the regions of* MMP*s 10, 14, and 16 are associated with dental caries.

## 2. Methods

### 2.1. Samples and Data Collection

Study participants were drawn from 6 parent studies in this investigation: The Center for Oral Health Research in Appalachia cohort 1 [COHRA1, *N* = 1,769 [39]], Iowa Head Start [IHS, *N* = 64 [6]], Iowa Fluoride Study [IFS, *N* = 136 [40, 41]], Dental Strategies Concentrating on Risk Evaluation [Dental SCORE, *N* = 502 [42, 43]], the Dental Registry and DNA Repository [DRDR, *N* = 875 [8]], and the Center for Education and Drug Abuse Research [CEDAR, *N* = 241 [44]] (Table 1).

All study protocols were approved by the Institutional Review Boards of the corresponding universities. Details of the participant recruitment protocol and study design for each parent study have been previously reported and summarized and further described in the Appendix (see Supplementary Material available online at https://doi.org/10.1155/2017/8465125). The 6 parent studies were stratified into 13 samples by age and race (8 adults and 5 children samples).

Dental caries assessments were performed by trained dental professionals (dentists or dental hygienists) via intraoral examination. Intraclass correlation coefficient (ICC) analysis was applied to measure the intra- and interexaminer reproducibility of the caries assessment. High concordance rates were observed for both interexaminer reliability (ICC = 0.86–0.99) and intraexaminer reliability (ICC > 0.99) [39]. Each tooth was identified as either permanent or primary and each surface on each tooth was scored for evidence of decay from which traditional DMFT and dft indices were generated. DMFT was defined as the number of decayed, missing due to decay, or restored (filled) teeth of the permanent dentition, excluding third molars. Correspondingly, dft was defined as the number of decayed or restored teeth of the primary dentition.

## 3. Genotypes

Genotyping for a custom panel of single nucleotide polymorphisms (SNPs) was performed by the Center for Inherited Disease Research (CIDR) using the Illumina GoldenGate platform (San Diego, USA). The majority of this panel was chosen to follow up results from GWAS scans of dental caries. Additionally, we also included SNPs such as those in and near MMP genes, based on our specific interest in strong candidate genes. For this study, we investigated three MMP genes:* MMP10*,* MMP14,* and* MMP16* (Table 2). These genes were selected based on their known roles in tooth development [32, 35, 38, 45] and previous investigations with dental caries [33]. The following procedure was used to select 28 tag SNPs within these genes for genotyping. We began with a list of all SNPs within a gene and filtered out variants with minor allele frequencies less than 2% because testing low-frequency polymorphisms was not feasible in our cohorts. We also filtered out variants with low “designability scores,” which is a measure of the predicted success of genotyping on the GoldenGate genotyping platform; we used 0.8 as the threshold in order to be reasonably certain that SNPs could be successfully genotyped. For the SNPs passing these filters we chose tag SNPs by pruning out SNPs with redundant information defined by multiple *R*^2^ < 0.95 in the reference CEU data (from the International HapMap Project), using a sliding window of up to 50 SNPs advancing one SNP at a time. The goal of this procedure was to capture much of the information on the genetic variation in a gene while genotyping the fewest number of individual SNPs. Note that technical limitations of the GoldenGate genotyping technology required that all SNPs be at least 60 base pairs away from each other. Overall, this SNP selection process yielded a set of low-correlated variants that were included based on their informativeness rather than their putative biological functions. Details regarding the design of the genotype panel are available elsewhere [46].

## 4. Statistical Analysis

Analyses of dental caries experience were performed separately in each sample for children 3–12 years of age for the primary dentition only (dft) and adults ≥ 18 years of age for the permanent dentition only (DMFT). Note, in children with mixed dentition, only the primary teeth present were included in the caries assessments because the goal was to identify risk factors influencing caries in the primary teeth in the child cohorts. The analyses were performed separately in self-reported non-Hispanics whites and blacks in order to guard against population stratification. Examinations of self-reported biological relationships among the participants were confirmed by genetic relatedness via 96 ancestry markers. Our CEDAR sample included adolescents > 15 years of age and for the purposes of this study was considered an adult sample. Linear regression analysis using PLINK software [47] was used to test genetic association between DMFT/dft and each SNP under the additive model while adjusting for age and sex. To guard against confounding due to admixture, we adjusted for the first 4 principal components for analyses of blacks. Further discussion of statistical analyses is presented in the Appendix.

Results were combined across samples using Stouffer's inverse variance weighted method of meta-analysis using METAL software [48]. This method is appropriate because it takes into consideration the nonrandom heterogeneity that is exhibited by the cohorts. Meta-analyses were performed for whites only and for all participants. Given the multiple comparisons, we used the method by Li and Ji [49] to determine the effective number of independent tests, which is less than or equal to the total number of tests due to linkage disequilibrium (LD; i.e., correlation) among SNPs. The threshold for declaring statistical significance was set to *p* value of 0.05, divided by the number of independent tests.

## 5. Results

**Figure 1:**
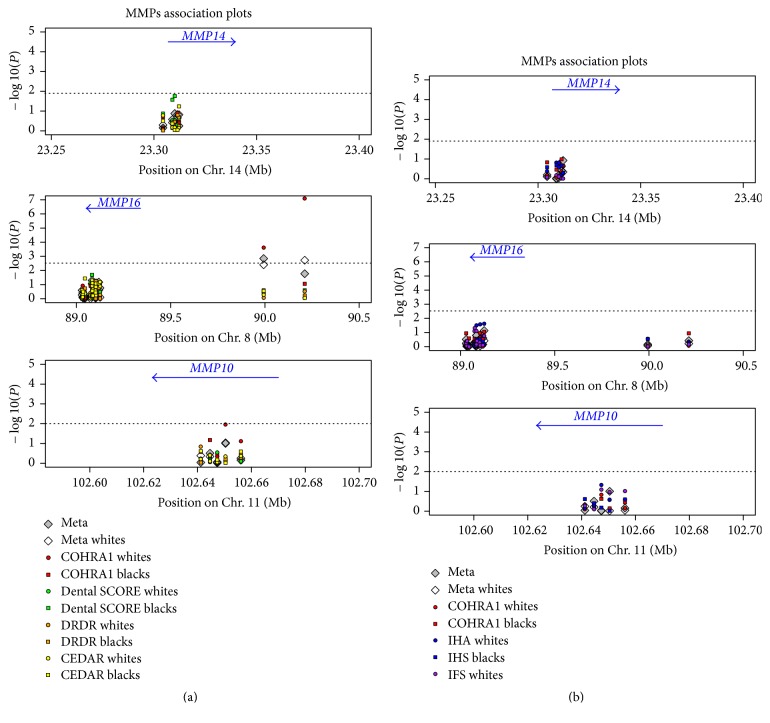
Genetic association in adults and children samples for 3 MMP genes. Genetic association in samples of adults (a) and children (b) for 3 MMP genes. Negative log_10_ transformed *p* values are shown. Childhood samples: Center for Oral Health in Appalachia (COHRA1 (red)), Iowa Head Start (IHS (blue)), and Iowa Fluoride Study (IFS (purple)). Adult samples: Center for Oral Health in Appalachia (COHRA1 (red)), Dental Strategies Concentrating on Risk Evaluation (Dental SCORE (green)), Dental Registry and DNA Repository (DRDR (orange)), and Center for Education and Drug Abuse Research (CEDAR (yellow)). Circles represent white samples, and squares represent black samples. For children samples, white diamonds represent meta-analysis across all white childhood samples, and gray diamonds represent meta-analysis across all black and white childhood samples combined. Similarly, for adult samples, white diamonds represent meta-analysis across all white adult samples and gray diamonds represent meta-analysis across all black and white adult samples, combined. The dotted lines represent the *p* threshold after adjustment for the number of independent single nucleotide polymorphisms within a gene. The physical location and directions of the genes are denoted by the blue arrows.

Characteristics of the 13 samples are shown in Table 1. There were noticeable differences in dental caries experience, which were expected because of the differences in age and demography within the samples.  Figure 1 shows the results of tests of genetic association for three matrix metalloproteinases genes:* MMP14*,* MMP16*, and* MMP10*. Negative log_10_-transformed *p* values are shown for tests of association in individual samples and combined. Detailed asscoation results for select SNPs from these genes are shown in the Appendix Table.

In adults, the strongest evidence of genetic association was detected for rs2046315, upstream of* MMP16* for COHRA1 whites (*p* = 8.14 × 10^−8^). In addition, meta-analysis across 8 white adult samples for this SNP yielded significant association as well (*p* = 0.002). SNP rs10429371 was significantly associated with caries for COHRA1 white adults (*p* = 0.0002) and via meta-analysis across black and white adults and children samples combined (*p* = 0.0005). Though not meeting the threshold after gene-wise adjustment for multiple comparisons, meta-analysis across white adults for rs10429371 showed nominal evidence of association (*p* = 0.004). Another convincing evidence of association that did not meet gene-wise threshold was for a SNP in* MMP10* for COHRA1 white adults (rs17293607, *p* = 0.01). There were no significant associations observed in any of the children samples.

## 6. Discussion

We investigated 28 SNPS in or near three* MMP* genes (*MMP10*,* MMP14*, and* MMP16*) for evidence of association with dental caries experience in 13 race- and age-stratified samples from 6 independent studies (*n* = 3600). Significant evidence of association was seen between two SNPs upstream of* MMP16* with dental caries in white adults and via meta-analysis across 8 adulthood samples.

Regarding the two SNPs upstream of* MMP16* associated in this study, noteworthy is SNP rs2046315. Our present work provided evidence that variant rs2046315, our strongest association (*p* = 8.14 × 10^−8^), is associated with dental caries in the permanent dentition for white adults. rs2046315 is located on chromosome 8q21.3 and is 870 kb upstream from* MMP16* and 560 kb away from the nearest gene* RIPK2* (receptor-interacting serine-threonine kinase 2).* RIKP2* has no known role in caries etiology, though it has been detected to be involved in apoptosis and is expressed in both deciduous and permanent tooth pulp cells [50]. Nearby rs10429371, also upstream of* MMP16*, showed evidence of association across multiple samples, though it was likewise driven by the COHRA1 white adults.* MMP16*, a member of the type I transmembrane proteins, has been suggested to be involved in early tooth development [38] and was relatively far (>100 kb) from our top SNP (rs2046315), and its role in caries etiology is unknown.

SNP rs2046315 was originally nominated in a GWAS scan of dental caries in a mostly overlapping sample of COHRA1 white adults [8]. In a subsequent GWAS of smooth surface caries in the permanent dentition, rs2046315 was also associated for increased caries susceptibility in COHRA1 adults (*p* = 3.08 × 10^−8^) [45]. Consistent with the previous findings, this SNP was suggestively associated with caries for the pit-and-fissures surface in the permanent dentition as well [45]. Therefore, in our study, we recapitulated the strong association observed in COHRA1 white adults but did not observe evidence of association in other samples for* MMP16*. Although* MMP16* is still a logical candidate to pursue, we did not strengthen evidence for its role in dental caries. In this study, we did not observe any associations for* MMP10 *and* MMP14* in either adults or children.

Potential reasons why we did not find any significant associations in samples other than COHRA1 whites was that heterogeneity was observed across our samples, such as associations that were specific to individual samples (i.e., COHRA1 white adults). This could be due to differences between populations, such as interactions with environmental factors that are present in specific populations but not in the others. Overall, matrix metalloproteinases genes are involved in numerous physiological processes and diseases such as dental caries. Though evidence of genetic association was not detected for* MMP10* and* MMP14*, results from our study suggest* MMP16* SNP rs2046315 may contribute to caries susceptibility in our individual sample of white adults. Even though rs2046315 was not proven to be a functional SNP, the multifactorial disorder of dental caries can involve several influential genes that potentially play a significant, but not an extensive role in the disease outcome. However, further investigation is needed to make this conclusion, and if truly associated, additional work is needed to determine the casual allele(s) and the mechanism impacting risk of caries.

## Supplementary Material

The appendix summarizes the participant recruitment protocol and study design for each parent study. In addition, the rational and description of our gene selection for our custom genotype panel, all statistical analyses performed and association results for MMP10, MMP14 and MMP16.

## Figures and Tables

**Table 1:** Characteristics of the samples: mean (range) or percentage, %.

Sample	*N*	Female sex	Age, years	dft/DMFT^1^
Children				
COHRA whites	608	46.7%	7.3 (3.0–12.0)	2.3 (0–17)
COHRA blacks	81	46.9%	7.6 (3.2–11.8)	1.8 (0–8)
IHS whites	41	58.5%	4.1 (3.2–5.3)	6.3 (0–20)
IHS blacks	23	52.2%	4.3 (3.4–5.6)	5.7 (0–17)
IFS whites	136	48.5%	5.2 (4.4–6.8)	1.2 (0–16)

Adults				
COHRA whites	994	62.8%	34.3 (18.0–75.0)	10.5 (0–28)
COHRA blacks	86	70.9%	36.2 (18.2–60.8)	9.3 (9–28)
Dental SCORE whites	277	63.2%	64.0 (48.0–78.0)	16.4 (2–28)
Dental SCORE blacks	225	72.9%	61.6 (47.0–79.0)	14.8 (1–28)
DRDR whites	702	50.0%	43.0 (18.0–74.8)	16.6 (0–28)
DRDR blacks	173	57.8%	44.5 (18.0–74.4)	16.5 (0–28)
CEDAR whites	173	31.2%	20.4 (15.7–28.6)	5.4 (0–21)
CEDAR blacks	68	44.3%	20.2 (15.6–27.8)	6.4 (0–16)

^1^dft was the measure of caries experience of the primary dentition in children samples; DMFT was the measure of caries experience of the permanent dentition in adult samples.

**Table 2:** Genetic variants in *MMPs* 10, 14, and 16.

SNP	Chromosome	Position^a^	MAF (COHRA1)^b^	Base change	Location/functionality
*MMP10*					
rs7948454	11	102641196	0.06	C-T	Downstream
rs12272341	11	102644601	0.13	A-G	Intronic
rs470154	11	102647310	0.06	C-G	Intronic
rs17293607	11	102650389	0.12	C-T	Missense Gly65Arg
rs559518	11	102656079	0.36	A-G	Intronic
*MMP14*					
rs8003217	14	23304416	0.16	A-C	Downstream
rs762052	14	23308986	0.14	A-G	Intronic
rs10133740	14	23310131	0.15	C-T	Intronic
rs17243048	14	23311480	0.17	A-G	Intronic
rs12893368	14	23312208	0.17	C-G	Intronic
*MMP16*					
rs17718917	8	89030490	0.08	A-G	Intergenic
rs1477907	8	89033615	0.09	A-G	Intergenic
rs16876790	8	89035664	0.38	A-T	Intergenic
rs2664368	8	89045674	0.20	C-T	Intergenic
rs10103111	8	89075226	0.23	C-T	Intronic
rs1824717	8	89075979	0.49	A-G	Intronic
rs17719876	8	89083319	0.08	C-T	Intronic
rs2616487	8	89084284	0.34	A-G	Intronic
rs6469206	8	89084691	0.45	G-T	Intronic
rs7826929	8	89084837	0.13	A-G	Intronic
rs2054415	8	89087358	0.05	G-T	Intronic
rs1551893	8	89102366	0.07	A-T	Intronic
rs1382104	8	89103325	0.44	C-T	Intronic
rs17720688	8	89104241	0.13	C-T	Intronic
rs10089111	8	89119305	0.38	G-T	Intronic
rs16878625	8	89125990	0.09	C-T	Intronic
rs10429371	8	89993488	0.22	C-T	Intergenic
rs2046315	8	90211100	0.13	A-G	Intergenic

^a^Based on Build 37, ^b^MAF = minor allele frequency in the COHRA1 sample.
