# Supplementary material for: Genetic Association of MMP10, MMP14, and MMP16 with Dental Caries
Source: Int J Dent. 2017 Feb 28;2017:8465125. doi: 10.1155/2017/8465125 (PMC5350286; doi:10.1155/2017/8465125)
Supplement: Supplementary file 1 — The appendix summarizes the participant recruitment protocol and study design for each parent study. In addition, the rational and description of our gene selection for our custom genotype panel, all statistical analyses performed and association results for MMP10, MMP14 and MMP16. [file 8465125.f1.docx]

APPENDIX

*Participant Recruitment*

**Center for Oral Health in Appalachia, cohort 1 (COHRA1)**

Families were recruited from two Pennsylvania counties (Washington and McKean) and two West Virginia counties (Webster and Nicholas) as part of a partnership between the University of Pittsburgh and West Virginia University to investigate factors (genetics, environmental, microbiological, and epidemiological) contributing to oral disease, oral health disparities, caries and other phenotypes in Appalachia. The eligibility criteria for recruitment included that at least one biological parent-child pair were included per household. Other members of eligible households were encouraged to participate regardless of biological relationships. Participants were enrolled without regard to their oral health status and were given complete intra-oral examinations by a licensed dentist or research dental hygienist. Details regarding the recruitment protocol are previously described [1]. DNA was isolated from saliva, blood, buccal swab and mouthwash samples using the Oragene kits from DNA Genotek (http://www.dnagenotek.com). Approval of this study population was obtained from the University of Pittsburgh and West Virginia University Institutional Review Board (IRB).

**Dental Strategies Concentrating On Risk Evaluation (SCORE)**

The Dental SCORE was established by the University of Pittsburgh to investigate the relationship between oral health and cardiovascular disease. This study derived from a prospective longitudinal cohort study called Heart SCORE designed to investigate the factors contributing to racial and socioeconomic disparities in cardiovascular risk in adults [2, 3]. African American and Caucasian adults within the Pittsburgh area who were already enrolled in the Heart SCORE study were asked to participate. All participants over the age of 45 were offered enrollment without regard to oral health status. Once participants provided informed consent, they received dental screening by a research dental hygienist following the COHRA1 protocol. DNA was isolated via salvia saliva samples from Oragene kits from DNA Genotek. All assessments were approved by the University of Pittsburgh IRB.

**Dental Registry and DNA Repository (DRDR)**

DRDR was established at the University of Pittsburgh School of Dental Medicine to obtain DNA samples from patients seeking treatment at the University of Pittsburgh School of Dental Medicine clinic. The purpose of this registry is to link dental phenotypes to DNA samples for educational and research involvement. Participants were enrolled without regard of their oral health and medical status. DNA was isolated via saliva samples taken in Oragene kits from DNA Genotek. All participants provided written consent to have their dental phenotypes and genetic data used for future research studies. Approval of this study population was obtained from the University of Pittsburgh IRB.

**Iowa Head Start (IHS) Study**

The IHS study recruited participants according to the University of Iowa Institutional Review Board (IRB) guidelines and informed consent was obtained. This study consisted of low-income children aged 3 to 5 years old who participated in federally funded child development program [4, 5]. Dental caries experience was performed by licensed dentist and DNA was extracted via buccal or saliva samples taken in Oragene kits from DNA Genotek.

**Iowa Fluoride Study (IFS)**

The objective of this study is to evaluate fluoride exposures from dietary and non-dietary sources and to associate fluoride exposure with dental caries and fluorosis. The IFS is a study that recruited new mothers and newborns from eight Iowa postpartum wards and followed their offspring from childhood to adulthood [6]. Trained dentists performed dental assessments during field examinations for children at ages 4 to 6 years. DNA was obtained from blood, buccal swab, or saliva samples as part of additional genetics study. All parents provided informed written consent, and all children provided verbal assent. All study questionnaires, procedures, and protocols were approved by the IRB at the University of Iowa.

**Center for Education and Drug Abuse Research (CEDAR)**

CEDAR recruited adolescent offspring of fathers with and without substance use disorder from the Pittsburgh area enrolled in a study of substance use risk factors. The purpose of this study is to investigate substance use risk factors in the father’s offspring from the ages of 10-12 years through 30 years of age [7]. Dental examinations were performed by calibrated dental hygienist at the University of Pittsburgh School of Dental Medicine. Blood samples and DNA aliquots were obtained from the NIDA Center for Genetic Studies. Lymphoblast cells lines established from blood samples at the NIDA Center for Genetic Studies (https://zork5/wustl/nida) and provided DNA for the present study. All of participants’ parents provided informed consent and all study questionnaires and protocols were approved by the University of Pittsburgh IRB.

**Custom Genotyping Panel**

All participants were genotyped for a custom panel of single nucleotide polymorphisms by the Center for Inherited Disease Research (CIDR) at Johns-Hopkins University using the Illumina GoldenGate platform (San Diego, USA). The custom panel consisted of tagging SNPs from 71 genes in addition to subset of several hundred specific SNPs of interest. The genes on the panel were chosen for different reasons, with the majority of genes chosen to follow-up results from GWAS studies for oral health phenotypes. Inclusion of genes was based on their location and/or linkage disequilibrium with associated variants in addition to biological relevance of the gene, previously reported experimental evidence, or a role in the etiology of the oral health phenotype.

SNPs on the panel were chosen per se to replicate previous associations, or to capture the genetic variation in genes of interest. The overall goal of this panel is for replication and fine mapping of previously associated loci for a variety of different oral health phenotypes, including dental caries. Design of the custom panel has been described previously [8].

Multidimensional scaling was performed in PLINK [9] to generate components of ancestry. Agreements between self-reported race/ethnicities were consistent with genetically determined ancestry. Examinations of self-reported biological relationships among the participants were confirmed by genetic relatedness. Additionally, self-reported sex matched with the genetic sex among participants. In conclusion, the previous mentioned checking procedures were used to verify the identity of the DNA samples.

**Statistical Analysis**

In this study, a quantitative trait analysis of data from 3,587 non-Hispanic white and non-Hispanic black participants was preformed, testing for association between 28 SNPs and dental caries in the primary and permanent dentitions. Analyses of dental caries experience in the primary dentition (dft) were investigated in children in 3-12 years of age, and in the permanent dentition (DMFT) in adults 18 years or older. One exception was the CEDAR sample, which included adolescents 15 years or older, who were considered adults for the purpose of the present study. The rational for stratifying by race was to guard against population stratification which can lead to false positive associations. The purpose for stratifying by age was because loci may potentially exhibit differential effects on caries experience of primary and permanent dentitions.

Linear regression was used to test for association for each SNP one at a time, coding genotypes as the number of rare alleles (0, 1, or 2), while including sex and age as covariates. Because of the probability for individuals of African ancestry exhibiting population structure/admixture, the first 4 components of ancestry were also included as covariates for association analyses of blacks. Stouffer’s inverse variance weighted method of meta-analysis was used to combine evidence of association across studies based on the sample size, direction of effect, and *p* value of the association test using the software tool METAL. This method was well suited for our study due to the differences in age ranges and phenotype distributions in the primary and permanent dentitions. Meta-analysis was performed for white children, black children, white adults, black adults, and all participants combined.

Because our COHRA1 samples are composed of individuals from households of rural communities, which included a number of relatives, we took into consideration the problem of non-independence among these participants. We addressed this issue by sub-setting the parents from children and analyzing them separately. Furthermore, we previously computed the genomic inflation factors (λ) for children and adults to determine if there were any systematic biases that may be present across our genetic association test results. COHRA1 children and adults exhibited a λ of approximately 1.05 and 1.00, respectively.

**APPENDIX REFERENCES**

1. Polk, D.E., et al., *Study protocol of the Center for Oral Health Research in Appalachia (COHRA) etiology study.* BMC Oral Health, 2008. **8**(18): p. 8-18.

2. Aiyer, A.N., et al., *Racial differences in coronary artery calcification are not attributed to differences in lipoprotein particle sizes: the Heart Strategies Concentrating on Risk Evaluation (Heart SCORE) Study.* Am Heart J, 2007. **153**(2): p. 328-34.

3. Aiyer, A.N., et al., *Predictors of significant short-term increases in blood pressure in a community-based population.* Am J Med, 2007. **120**(11): p. 960-7.

4. Slayton, R.L., M.E. Cooper, and M.L. Marazita, *Tuftelin, mutans streptococci, and dental caries susceptibility.* J Dent Res, 2005. **84**(8): p. 711-4.

5. Marshall, T.A., et al., *Relative validity of the Iowa Fluoride Study targeted nutrient semi-quantitative questionnaire and the block kids' food questionnaire for estimating beverage, calcium, and vitamin D intakes by children.* J Am Diet Assoc, 2008. **108**(3): p. 465-72.

6. Wang, X., et al., *Genetic and environmental factors associated with dental caries in children: the Iowa Fluoride Study.* Caries Res, 2012. **46**(3): p. 177-84.

7. Vanyukov, M.M., et al., *Haplotypes of the monoamine oxidase genes and the risk for substance use disorders.* Am J Med Genet B Neuropsychiatr Genet, 2004. **125b**(1): p. 120-5.

8. Stanley, B.O., et al., *Genetic Association of MPPED2 and ACTN2 with Dental Caries.* J Dent Res, 2014. **93**(7): p. 626-632.

9. Purcell, S., et al., *PLINK: a tool set for whole-genome association and population-based linkage analyses.* Am J Hum Genet, 2007. **81**(3): p. 559-75.

**Appendix Table:** Beta-coefficients (p-values) of genetic association tests for select SNPs of *MMP10*, *MMP14* and *MMP16*.

| Gene | Children |  | Beta |  |  |  |  | P -values |  |  |  | Children | Beta | P -values |  |  | All |
| --- | --- | --- | --- | --- | --- | --- | --- | --- | --- | --- | --- | --- | --- | --- | --- | --- | --- |
|  | Whites |  |  |  |  |  |  |  |  |  |  | Blacks |  |  |  |  | Children |
| SNP | Chr | Position | COHRA | IHS | IFS |  | COHRA | IHS | IFS | Meta |  | COHRA | IHS | COHRA | IHS | Meta | Meta (All) |
|  |  | (BP) | N=608 | N=41 | N=136 |  |  |  |  | N=785 |  | N=81 | N=23 |  |  | N=104 | N=889 |
| MMP10 |  |  |  |  |  |  |  |  |  |  |  |  |  |  |  |  |  |
| rs7948454 | 11 | 102641196 | -0.09 | -1.62 | -0.33 |  | 0.80 | 0.69 | 0.53 | 0.56 |  | 0.37 | -1.67 | 0.49 | 0.55 | 0.53 | 0.98 |
| rs12272341 | 11 | 102644601 | 1.13 | 1.30 | -0.11 |  | 0.61 | 0.52 | 0.79 | 0.62 |  | -0.38 | -1.81 | 0.50 | 0.40 | 0.28 | 0.88 |
| rs470154 | 11 | 102647310 | -0.53 | 4.47 | 1.22 |  | 0.14 | 0.04 | 0.08 | 0.92 |  | 2.12 | -5.12 | 0.23 | 0.39 | 0.56 | 0.87 |
| rs17293607 | 11 | 102650389 | 0.26 | -2.04 | -0.52 |  | 0.27 | 0.26 | 0.11 | 0.96 |  | 0.33 | -2.17 | 0.72 | 0.56 | 0.76 | 0.76 |
| rs559518 | 11 | 102656079 | -0.06 | -1.26 | 0.53 |  | 0.70 | 0.25 | 0.09 | 0.91 |  | -0.36 | -2.51 | 0.35 | 0.31 | 0.92 | 0.89 |
| MMP14 |  |  |  |  |  |  |  |  |  |  |  |  |  |  |  |  |  |
| rs8003217 | 14 | 23304416 | -0.10 | -1.35 | 0.07 |  | 0.67 | 0.41 | 0.84 | 0.63 |  | 0.80 | 3.32 | 0.14 | 0.26 | 0.70 | 0.77 |
| rs762052 | 14 | 23308986 | 0.04 | -2.45 | 0.15 |  | 0.86 | 0.15 | 0.66 | 0.99 |  | 0.61 | 3.05 | 0.34 | 0.20 | 0.86 | 0.94 |
| rs10133740 | 14 | 23310131 | 0.09 | -2.46 | 0.09 |  | 0.69 | 0.14 | 0.77 | 0.89 |  | 0.71 | 2.75 | 0.21 | 0.25 | 0.41 | 0.65 |
| rs17243048 | 14 | 23311480 | 0.18 | 0.74 | 0.02 |  | 0.43 | 0.63 | 0.95 | 0.40 |  | 1.26 | -3.32 | 0.10 | 0.21 | 0.27 | 0.23 |
| rs12893368 | 14 | 23312208 | 0.15 | 1.01 | 0.01 |  | 0.49 | 0.53 | 0.97 | 0.45 |  | 0.70 | -1.90 | 0.22 | 0.51 | 0.02 | 0.11 |
| MMP16 |  |  |  |  |  |  |  |  |  |  |  |  |  |  |  |  |  |
| rs17718917 | 8 | 89030490 | -0.20 | -1.52 | 0.26 |  | 0.50 | 0.44 | 0.58 | 0.58 |  | -1.37 | 6.66 | 0.48 | 0.29 | 0.19 | 0.31 |
| rs1477907 | 8 | 89033615 | -0.14 | 0.06 | 0.07 |  | 0.63 | 0.97 | 0.87 | 0.72 |  | 0.23 | 1.40 | 0.60 | 0.44 | 0.37 | 0.97 |
| rs16876790 | 8 | 89035664 | -0.48 | 0.01 | 0.23 |  | 0.79 | 0.98 | 0.40 | 0.90 |  | 0.10 | -1.89 | 0.77 | 0.41 | 0.64 | 0.93 |
| rs2664368 | 8 | 89045674 | -0.05 | 0.36 | 0.01 |  | 0.81 | 0.82 | 0.99 | 0.88 |  | -0.42 | 0.01 | 0.26 | 0.99 | 0.55 | 0.92 |
| rs10103111 | 8 | 89075226 | 0.07 | -0.76 | -0.57 |  | 0.73 | 0.61 | 0.07 | 0.58 |  | -0.48 | -0.82 | 0.27 | 0.71 | 0.64 | 0.49 |
| rs1824717 | 8 | 89075979 | 0.13 | -0.38 | -0.53 |  | 0.46 | 0.74 | 0.04 | 0.79 |  | -0.15 | 0.16 | 0.69 | 0.92 | 0.49 | 0.61 |
| rs17719876 | 8 | 89083319 | -0.01 | 3.82 | 0.08 |  | 0.23 | 0.02 | 0.83 | 0.64 |  | 0.61 | NA | 0.67 | NA | 0.53 | 0.85 |
| rs2616487 | 8 | 89084284 | 0.09 | 0.17 | 0.12 |  | 0.62 | 0.91 | 0.69 | 0.53 |  | 0.55 | NA | 0.49 | NA | 0.46 | 0.39 |
| rs6469206 | 8 | 89084691 | -0.03 | -2.65 | 0.11 |  | 0.85 | 0.03 | 0.70 | 0.62 |  | -0.01 | -1.77 | 0.96 | 0.04 | 0.48 | 0.86 |
| rs7826929 | 8 | 89084837 | 0.20 | -0.23 | 0.74 |  | 0.42 | 0.88 | 0.07 | 0.16 |  | -0.01 | -2.53 | 0.01 | 0.32 | 0.76 | 0.16 |
| rs2054415 | 8 | 89087358 | -0.00 | -2.53 | 0.76 |  | 0.92 | 0.66 | 0.39 | 0.70 |  | -2.58 | NA | 0.31 | NA | 0.26 | 0.43 |
| rs1551893 | 8 | 89102366 | -0.31 | -2.90 | 0.43 |  | 0.37 | 0.32 | 0.51 | 0.46 |  | 0.17 | -0.79 | 0.78 | 0.80 | 0.68 | 0.40 |
| rs1382104 | 8 | 89103325 | 0.29 | -1.14 | -0.06 |  | 0.11 | 0.30 | 0.82 | 0.28 |  | -0.48 | -0.79 | 0.31 | 0.82 | 0.81 | 0.28 |
| rs17720688 | 8 | 89104241 | 0.25 | 1.65 | 0.42 |  | 0.23 | 0.02 | 0.83 | 0.64 |  | 1.41 | NA | 0.29 | NA | 0.29 | 0.90 |
| rs10089111 | 8 | 89119305 | 0.01 | -1.86 | -0.08 |  | 0.96 | 0.18 | 0.77 | 0.69 |  | -0.49 | -1.46 | 0.27 | 0.43 | 0.04 | 0.25 |
| rs16878625 | 8 | 89125990 | 0.07 | -5.20 | 0.49 |  | 0.80 | 0.02 | 0.17 | 0.38 |  | -1.07 | 2.44 | 0.08 | 0.67 | 0.01 | 0.07 |
| rs10429371 | 8 | 89993488 | -0.02 | 1.93 | 0.01 |  | 0.92 | 0.27 | 0.95 | 0.85 |  | -0.09 | -2.50 | 0.82 | 0.28 | 0.57 | 0.69 |
| rs2046315 | 8 | 90211100 | -0.05 | -1.23 | -0.14 |  | 0.02 | 0.21 | 0.32 | 0.07 |  | -0.66 | 1.24 | 0.11 | 0.58 | 0.28 | 0.37 |

|  | Adults |  |  |  | Beta |  |  |  |  | P-values |  |  |  |  | Adults | Beta |  |  |  |  | P-values | |  | All Adults | Children + Adults |
| --- | --- | --- | --- | --- | --- | --- | --- | --- | --- | --- | --- | --- | --- | --- | --- | --- | --- | --- | --- | --- | --- | --- | --- | --- | --- |
| Gene | Whites |  |  |  |  |  |  |  |  | Whites |  |  |  |  | Blacks |  |  |  |  |  |  |  |  |  |  |
| SNP | Chr | Position | COHRA |  | Dental SCORE | DRDR | CEDAR |  | COHRA | Dental SCORE | DRDR | CEDAR | Meta |  | COHRA | Dental SCORE | DRDR | CEDAR | COHRA | Dental Score | DRDR | CEDAR | Meta | Meta | Meta |
|  |  |  | N=994 |  | N=277 | N=702 | N=173 |  |  |  |  |  | N=2,146 |  | N=86 | N=225 | N=173 | N=68 |  |  |  |  | N=552 | N=2,698 | N=3,587 |
| MMP10 |  |  |  |  |  |  |  |  |  |  |  |  |  |  |  |  |  |  |  |  |  |  |  |  |  |
| rs7948454 | 11 | 102641196 | -0.07 |  | 0.46 | 1.09 | -0.54 |  | 0.90 | 0.57 | 0.14 | 0.61 | 0.41 |  | -1.39 | 0.29 | 0.08 | -1.29 | 0.26 | 0.65 | 0.91 | 0.24 | 0.62 | 0.61 | 0.91 |
| rs12272341 | 11 | 102644601 | -0.08 |  | -0.43 | -0.45 | 0.20 |  | 0.86 | 0.50 | 0.43 | 0.80 | 0.45 |  | -2.17 | -0.29 | 0.25 | -0.99 | 0.06 | 0.67 | 0.81 | 0.39 | 0.24 | 0.23 | 0.31 |
| rs470154 | 11 | 102647310 | -0.43 |  | 1.03 | 0.01 | -0.14 |  | 0.45 | 0.28 | 0.99 | 0.88 | 0.86 |  | 4.28 | -0.65 | 0.12 | -2.02 | 0.35 | 0.75 | 0.96 | 0.66 | 0.96 | 0.89 | 0.97 |
| rs17293607 | 11 | 102650389 | 1.02 |  | -0.11 | 0.22 | -0.55 |  | 0.01 | 0.84 | 0.71 | 0.45 | 0.09 |  | 1.50 | 0.37 | 0.96 | 0.16 | 0.61 | 0.74 | 0.62 | 0.95 | 0.48 | 0.06 | 0.09 |
| rs559518 | 11 | 102656079 | 0.29 |  | 0.45 | 0.42 | 0.54 |  | 0.07 | 0.41 | 0.33 | 0.43 | 0.56 |  | 0.24 | -0.13 | -0.49 | 0.85 | 0.81 | 0.80 | 0.52 | 0.25 | 0.98 | 0.59 | 0.69 |
| MMP14 |  |  |  |  |  |  |  |  |  |  |  |  |  |  |  |  |  |  |  |  |  |  |  |  |  |
| rs8003217 | 14 | 23304416 | 0.16 |  | -0.17 | 0.09 | 1.01 |  | 0.67 | 0.76 | 0.85 | 0.16 | 0.51 |  | -1.64 | 1.01 | -0.05 | -1.19 | 0.19 | 0.13 | 0.94 | 0.31 | 0.95 | 0.54 | 0.70 |
| rs762052 | 14 | 23308986 | 0.41 |  | 0.64 | 0.53 | 0.73 |  | 0.36 | 0.29 | 0.37 | 0.67 | 0.38 |  | -1.08 | 1.75 | 0.37 | -0.87 | 0.46 | 0.02 | 0.71 | 0.42 | 0.29 | 0.20 | 0.26 |
| rs10133740 | 14 | 23310131 | 0.38 |  | -0.67 | 0.49 | 0.31 |  | 0.34 | 0.29 | 0.35 | 0.68 | 0.35 |  | -0.57 | 1.78 | 0.24 | -0.13 | 0.69 | 0.01 | 0.80 | 0.90 | 0.14 | 0.14 | 0.13 |
| rs17243048 | 14 | 23311480 | -0.28 |  | -0.24 | -0.74 | 0.10 |  | 0.45 | 0.64 | 0.15 | 0.86 | 0.15 |  | -2.95 | -0.65 | -2.46 | -0.17 | 0.12 | 0.50 | 0.15 | 0.89 | 0.06 | 0.03 | 0.21 |
| rs12893368 | 14 | 23312208 | -0.35 |  | -0.23 | -0.65 | 0.23 |  | 0.33 | 0.66 | 0.20 | 0.70 | 0.15 |  | -1.11 | -0.76 | -0.61 | 1.73 | 0.42 | 0.24 | 0.58 | 0.05 | 0.48 | 0.11 | 0.56 |
| MMP16 |  |  |  |  |  |  |  |  |  |  |  |  |  |  |  |  |  |  |  |  |  |  |  |  |  |
| rs17718917 | 8 | 89030490 | -0.16 |  | -0.87 | 0.01 | -0.15 |  | 0.75 | 0.30 | 0.98 | 0.88 | 0.55 |  | 0.03 | -0.47 | 0.75 | -5.59 | 0.99 | 0.76 | 0.76 | 0.24 | 0.67 | 0.85 | 0.84 |
| rs1477907 | 8 | 89033615 | 0.74 |  | -0.07 | -0.28 | 0.29 |  | 0.11 | 0.89 | 0.67 | 0.75 | 0.38 |  | -0.05 | -0.67 | -0.76 | 0.74 | 0.95 | 0.24 | 0.37 | 0.44 | 0.31 | 0.75 | 0.77 |
| rs16876790 | 8 | 89035664 | 0.04 |  | 0.32 | -0.17 | -0.53 |  | 0.88 | 0.45 | 0.67 | 0.35 | 0.89 |  | 0.52 | -0.15 | 0.26 | -0.86 | 0.63 | 0.76 | 0.72 | 0.31 | 0.87 | 0.85 | 0.84 |
| rs2664368 | 8 | 89045674 | -0.14 |  | -0.30 | 0.46 | 0.54 |  | 0.67 | 0.52 | 0.34 | 0.43 | 0.81 |  | -0.12 | -0.30 | -1.03 | 1.55 | 0.90 | 0.56 | 0.18 | 0.03 | 0.66 | 0.98 | 0.94 |
| rs10103111 | 8 | 89075226 | -0.19 |  | -0.13 | 0.44 | -0.05 |  | 0.57 | 0.78 | 0.37 | 0.92 | 0.99 |  | -1.57 | -0.13 | 0.07 | 0.43 | 0.10 | 0.80 | 0.92 | 0.61 | 0.56 | 0.79 | 0.56 |
| rs1824717 | 8 | 89075979 | -0.22 |  | -0.31 | -0.08 | 0.44 |  | 0.42 | 0.44 | 0.84 | 0.40 | 0.48 |  | -1.41 | 0.90 | 0.04 | -0.45 | 0.12 | 0.07 | 0.94 | 0.55 | 0.69 | 0.65 | 0.51 |
| rs17719876 | 8 | 89083319 | -0.04 |  | -0.38 | -1.39 | -1.40 |  | 0.92 | 0.63 | 0.06 | 0.12 | 0.08 |  | 1.04 | -4.89 | -3.15 | 1.01 | 0.78 | 0.02 | 0.22 | 0.64 | 0.05 | 0.01 | 0.05 |
| rs2616487 | 8 | 89084284 | 0.32 |  | 0.21 | -0.72 | 1.14 |  | 0.28 | 0.63 | 0.07 | 0.04 | 0.70 |  | -1.90 | -0.64 | -0.47 | 0.50 | 0.40 | 0.48 | 0.72 | 0.74 | 0.39 | 0.95 | 0.69 |
| rs6469206 | 8 | 89084691 | -0.03 |  | 0.02 | 0.20 | 0.52 |  | 0.89 | 0.95 | 0.61 | 0.31 | 0.62 |  | 1.10 | 0.17 | -0.37 | 0.85 | 0.23 | 0.72 | 0.65 | 0.27 | 0.41 | 0.42 | 0.54 |
| rs7826929 | 8 | 89084837 | -0.45 |  | 0.04 | -0.30 | 1.48 |  | 0.27 | 0.94 | 0.62 | 0.07 | 0.59 |  | 1.15 | 0.41 | -0.64 | 0.40 | 0.28 | 0.51 | 0.41 | 0.64 | 0.58 | 0.82 | 0.60 |
| rs2054415 | 8 | 89087358 | -0.80 |  | 0.30 | -0.39 | 1.76 |  | 0.24 | 0.77 | 0.65 | 0.22 | 0.53 |  | 2.94 | 1.02 | 0.92 | -2.35 | 0.44 | 0.59 | 0.69 | 0.60 | 0.49 | 0.80 | 0.54 |
| rs1551893 | 8 | 89102366 | 0.24 |  | 0.68 | -0.27 | -1.17 |  | 0.66 | 0.34 | 0.73 | 0.25 | 0.89 |  | 3.11 | 0.15 | 0.03 | -1.24 | 0.05 | 0.89 | 0.97 | 0.36 | 0.58 | 0.70 | 0.91 |
| rs1382104 | 8 | 89103325 | -0.08 |  | -0.22 | 0.03 | -0.85 |  | 0.77 | 0.58 | 0.92 | 0.08 | 0.42 |  | -1.35 | 0.29 | 1.74 | 1.31 | 0.30 | 0.64 | 0.05 | 0.14 | 0.14 | 0.96 | 0.60 |
| rs17720688 | 8 | 89104241 | -0.20 |  | -0.10 | -0.24 | 0.50 |  | 0.63 | 0.86 | 0.67 | 0.56 | 0.63 |  | 0.89 | 3.19 | 2.08 | NA | 0.78 | 0.12 | 0.25 | NA | 0.06 | 0.71 | 0.80 |
| rs10089111 | 8 | 89119305 | -0.09 |  | -0.05 | -0.45 | -0.21 |  | 0.75 | 0.91 | 0.25 | 0.68 | 0.30 |  | -1.13 | -0.72 | 1.01 | -1.49 | 0.25 | 0.16 | 0.17 | 0.08 | 0.24 | 0.14 | 0.06 |
| rs16878625 | 8 | 89125990 | -0.10 |  | 0.80 | -0.40 | 1.29 |  | 0.83 | 0.32 | 0.53 | 0.12 | 0.80 |  | 0.20 | -1.22 | 0.03 | -2.13 | 0.90 | 0.10 | 0.97 | 0.06 | 0.10 | 0.60 | 0.17 |
| rs10429371 | 8 | 89993488 | 1.29 |  | 0.46 | -0.08 | 0.41 |  | *.0002 | 0.38 | 0.84 | 0.53 | .004 |  | 0.78 | 0.57 | 0.94 | 0.90 | 0.44 | 0.28 | 0.25 | 0.28 | 0.04 | *.0005 | *.0001 |
| rs2046315 | 8 | 90211100 | 2.25 |  | 0.11 | -0.60 | -0.13 |  | *8.14E^-8^ | 0.85 | 0.30 | 0.86 | *0.002 |  | 1.66 | 0.65 | -0.41 | 0.12 | 0.08 | 0.25 | 0.60 | 0.87 | 0.24 | *9.8E^-4^ | 0.01 |

*Indicates statistically significant p-values after gene-wise adjustment for multiple comparisons. COHRA, Center for Oral Health in Appalachia; IHS, Iowa Head Start; IFS, Iowa Fluoride Study; Dental SCORE, Dental Strategies Concentrating on Risk Evaluation; DRDR, Dental Registry and DNA Repository; CEDAR, Center for Education and Drug Abuse Research.
